# Work Does Not End When You Leave: Concerns and Coping Strategies Among Nursing Home Social Workers During COVID-19

**DOI:** 10.1093/geroni/igab046.1474

**Published:** 2021-12-17

**Authors:** Noelle Fields, Vivian Miller, Keith Anderson, Nancy Kusmaul

**Affiliations:** 1 University of Texas at Arlington, Arlington, Texas, United States; 2 Bowling Green State University, Bowling Green, Ohio, United States; 3 University of Maryland, Baltimore County, Baltimore, Maryland, United States

## Abstract

Nursing home social workers (NH SW) at the frontline during COVID-19 are faced with many challenges in meeting the psychosocial needs of residents while balancing their own well-being needs. In order to explore the experiences of NH SW during COVID-19, the study utilized a cross-sectional survey distributed to social media sites (e.g., Reddit, Facebook) and professional networks. The survey asked participants (N = 63) open-ended questions which were analyzed using the rigorous and accelerated data reduction (RADaR) method. Themes suggested that fear for self, lack of administrative support, and overall stress were notable concerns among NH SW. Findings also suggested that support from family/friends and self-care were most personally helpful to NH SW. Lastly, themes related to coping strategies included talking with co-workers, mindfulness, and boundary setting. Findings suggest the need for increased supports for NH SW. Implications related to stress and coping during COVID-19 are offered.

